# Case Report: Delayed intra-abdominal hemorrhage in a patient with a duodenal stump tumor after Billroth II gastrectomy

**DOI:** 10.3389/fonc.2026.1739532

**Published:** 2026-04-21

**Authors:** Bin Wu

**Affiliations:** Department of Hepatobiliary Surgery, the Second Affiliated Hospital of Jiaxing University, Jiaxing, China

**Keywords:** digital subtraction angiography, duodenal adenocarcinoma, duodenal stump tumor, intra-abdominal hemorrhage, postoperative complication, pseudoaneurysm

## Abstract

**Background** Duodenal tumors are rare, and those originating from the duodenal stump (the blind end of the duodenum remaining after gastrectomy with Billroth II anastomosis) are even rarer.

**Methods** We report a case of a patient with duodenal stump adenocarcinoma who had a 30-year history of subtotal gastrectomy with Billroth II anastomosis. The patient presented with chronic gastrointestinal bleeding and secondary anemia, prompting tumor resection and lymph node dissection. However, on postoperative day 11, she developed delayed intra-abdominal hemorrhage due to a pseudoaneurysm in a minor branch of the superior mesenteric artery. Despite aggressive management including DSA-guided arterial embolization, the patient ultimately had a poor prognosis due to subsequent ischemic colitis. The rarity of duodenal stump tumors and the uncommon occurrence of delayed postoperative pseudoaneurysm rupture make this case clinically notable, highlighting key challenges in diagnosis, surgical management, and complication mitigation.

**Findings:**

A 77-year-old female with a 3-month history of dizziness and fatigue was diagnosed with massive gastrointestinal hemorrhage and duodenal stump adenocarcinoma via multimodal evaluation. She underwent duodenal stump tumor resection. On postoperative day 11, she developed delayed intra-abdominal hemorrhage from a artery pseudoaneurysm. Abdominal CTA detected a pseudoaneurysm in a superior mesenteric artery branch with contrast extravasation; emergency DSA-guided embolization successfully stopped bleeding. However, on postoperative day 25, she developed severe rectal bleeding; colonoscopy showed extensive ischemic necrosis in the descending colon. Despite supportive care, the patient’s family discontinued treatment due to persistent bleeding and septic shock, and she died 1 week after discharge.

**Interpretation:**

Duodenal stump tumors require a high index of suspicion for diagnosis, as their nonspecific symptoms are often misattributed to postsurgical sequelae. Multimodal imaging and gastroscopy with biopsy are critical for preoperative planning. Delayed postoperative pseudoaneurysm-related hemorrhage is extremely rare but life-threatening; CTA and DSA are essential for rapid diagnosis and intervention. While DSA-guided embolization effectively controls bleeding, it carries a risk of bowel ischemia that requires close monitoring. This case underscores the need for meticulous preoperative assessment, careful surgical technique to avoid vascular injury, and postoperative vigilance for rare complications—supporting the utility of integrated clinical-radiological management in complex cases.

## Highlights

- Evidence before this study
  Duodenal tumors are rare, and duodenal stump tumors post-Billroth II gastrectomy are even rarer, with only scattered case reports. They often present with nonspecific symptoms (chronic anemia, gastrointestinal bleeding) easily misdiagnosed as postsurgical complications. Enhanced CT/MRI aid in preoperative evaluation, but gastroscopy (gold standard for biopsy) is technically challenging for Billroth II patients. Surgical resection is primary treatment—pancreatoduodenectomy for advanced tumors (high morbidity) and limited resection for small lesions (scarce evidence). Delayed intra-abdominal hemorrhage from mesenteric pseudoaneurysms is extremely rare, with unclear pathogenesis; DSA embolization is effective but has unaddressed ischemia risks.
- Added value of this study
  This case details a 77-year-old with duodenal stump adenocarcinoma who had delayed hemorrhage from a superior mesenteric pseudoaneurysm. It confirms multimodal imaging (CT/MRI/CTA) for preoperative tumor evaluation and postoperative complication diagnosis. It verifies limited resection’s feasibility for small tumors (negative margins, smooth early recovery) and provides practical experience for DSA embolization. It also highlights a rare sequela—descending colon ischemia post-embolization, reminding clinicians of intestinal blood supply monitoring.
- Implications of all the available evidence
  Clinicians need high suspicion for duodenal stump tumors in post-gastrectomy patients with unexplained anemia/bleeding, using multimodal imaging and gastroscopy. Individualize surgery: limited resection for small tumors, pancreatoduodenectomy for advanced ones. Monitor for rare delayed hemorrhage—use CTA for diagnosis and DSA for embolization, with close post-embolization intestinal ischemia surveillance. More cohort studies are needed to establish standardized guidelines for this rare disease.

## Introduction

Duodenal cancer is a rare gastrointestinal malignancy, with an incidence of ~0.3–0.5 per 100, 000 individuals annually (1). Tumors arising from the duodenal stump—defined as the residual blind end of the duodenum after subtotal gastrectomy with Billroth II anastomosis—are even rarer, representing <1% of all duodenal neoplasms (5). These tumors pose unique challenges due to their anatomical location (adjacent to the pancreas, bile duct, and superior mesenteric vessels) and altered postoperative anatomy, which complicate diagnosis and surgical access (3).

Patients with duodenal stump tumors often present with nonspecific symptoms, such as chronic anemia or intermittent gastrointestinal bleeding, which are frequently attributed to postsurgical complications (e.g., anastomotic ulcers) (6)—leading to delayed diagnosis. Surgical resection remains the only curative treatment, but the procedure is technically demanding due to adhesions from previous surgery and proximity to critical structures (12). Postoperative complications such as infection or pancreatic fistula are well recognized, but delayed intra-abdominal hemorrhage due to pseudoaneurysm formation is extremely rare (15), with no standardized management guidelines.

We report a case of a 77-year-old female with duodenal stump adenocarcinoma who developed a pseudoaneurysm of a superior mesenteric artery branch on postoperative day 11, followed by ischemic colitis after embolization.

## Methods

### Case presentation

#### Chief complaints

A 77-year-old elderly female patient presented to our hospital with dizziness and fatigue that had lasted for three months.

#### History of present illness

Three months prior, the patient began to experience dizziness and fatigue and was diagnosed with “anemia” at a local hospital. It was suggested that she undergo gastroscopy, but the patient was unwilling to undergo the examination and was instead given iron supplementation and other treatments.The patient’s symptoms showed no improvement. Three months after symptom onset, gastroscopy performed at Pinghu First People’s Hospital indicated “gastrointestinal bleeding, suspected bleeding from the afferent loop after gastrectomy.”

#### History of past illness

The patient had a 20-year history of hypertension and had been taking amlodipine 5 mg orally once daily for long-term blood pressure control. Before admission, her blood pressure was generally controlled at 130–150/60–70 mmHg, with suboptimal control. She had undergone subtotal gastrectomy with Billroth II anastomosis more than 30 years prior; the indication for the original gastrectomy was unknown, and the patient denied a history of gastric tumor.

#### Physical examination

Physical examination on admission revealed the following: body temperature 36.4 °C, pulse 83 beats/min, respiratory rate 19 breaths/min, blood pressure 143/60 mmHg. The patient appeared pale, with no jaundice of the skin or sclera. The abdomen was soft, non-tender, with no palpable mass, hepatosplenomegaly, or shifting dullness. Bowel sounds were 4 times per minute. No lower extremity edema was noted.

#### Laboratory examinations

**Figure 1:**
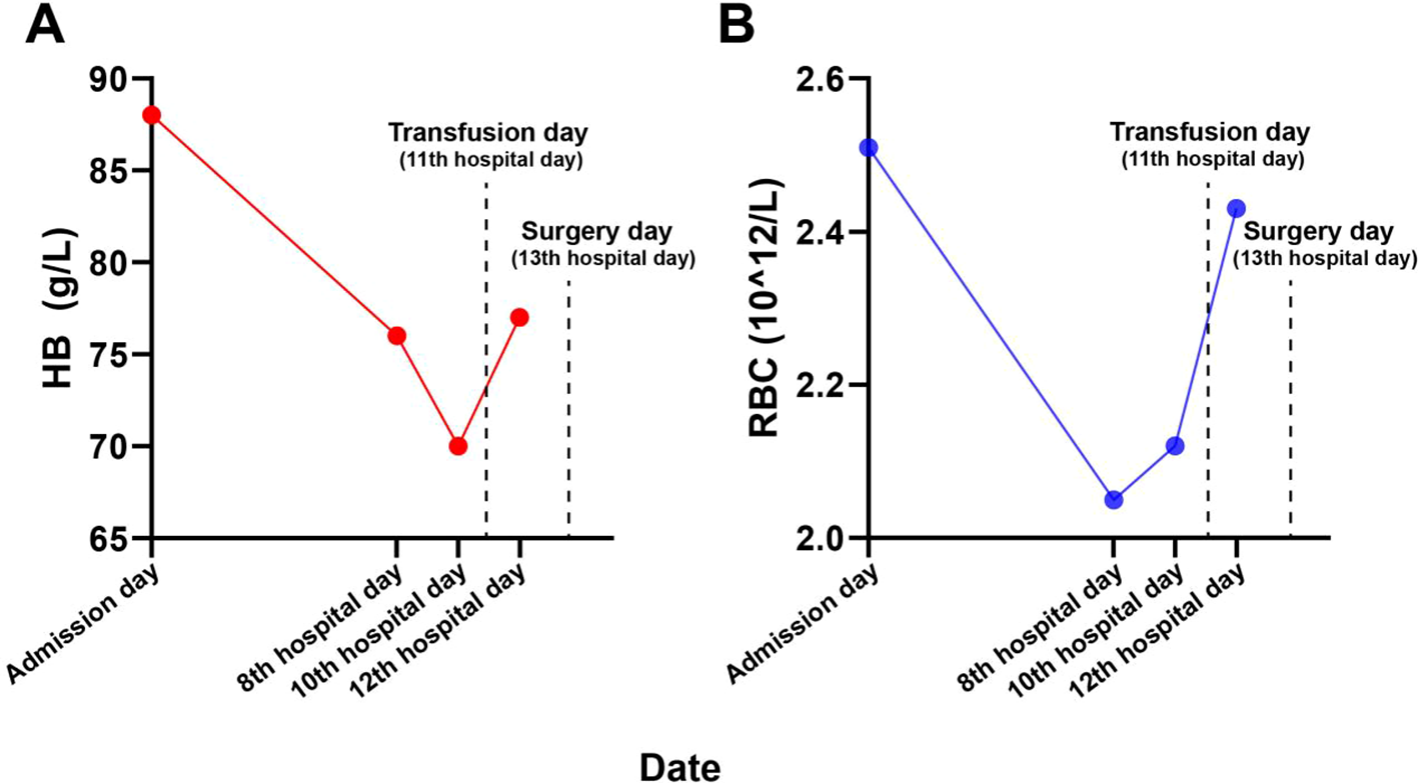
Shows the changes in red blood cell count **(A)** and hemoglobin level **(B)** before surgery.

Upon admission, the patient’s hemoglobin was 88 g/L, her red blood cell count was 2.51×10^12/L, her reticulocyte percentage was 13.37%, and her reticulocyte count was 275.4×10^9/L. However, during hospitalization, the hemoglobin levels gradually decreased (**Figure 1**). Before preoperative transfusion, hemoglobin decreased to 70 g/L and red blood cell count decreased to 2.05 g/L. Hemoglobin and red blood cell counts were elevated following packed red blood cell transfusion. The patient’s coagulation profile was normal.

#### Imaging examinations

**Figure 2:**
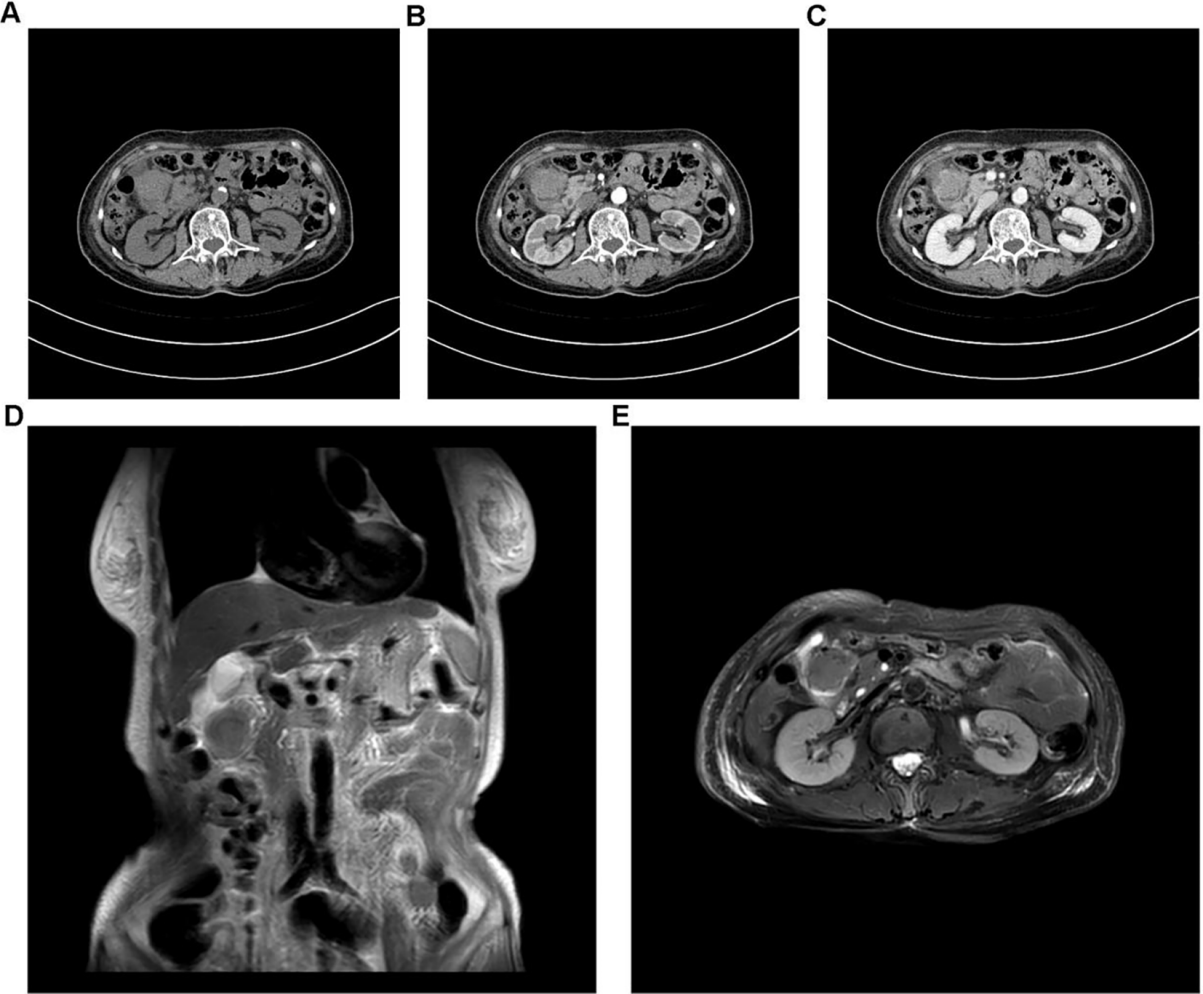
Imaging studies of the duodenal stump tumor. **(A)** Plain scan of the abdominal enhanced CT; **(B)** Arterial phase of the abdominal enhanced CT; **(C)** Venous phase of the abdominal enhanced CT; **(D)** Coronal section of the MRI; **(E)** Transverse section of the MRI.

**Figure 3:**
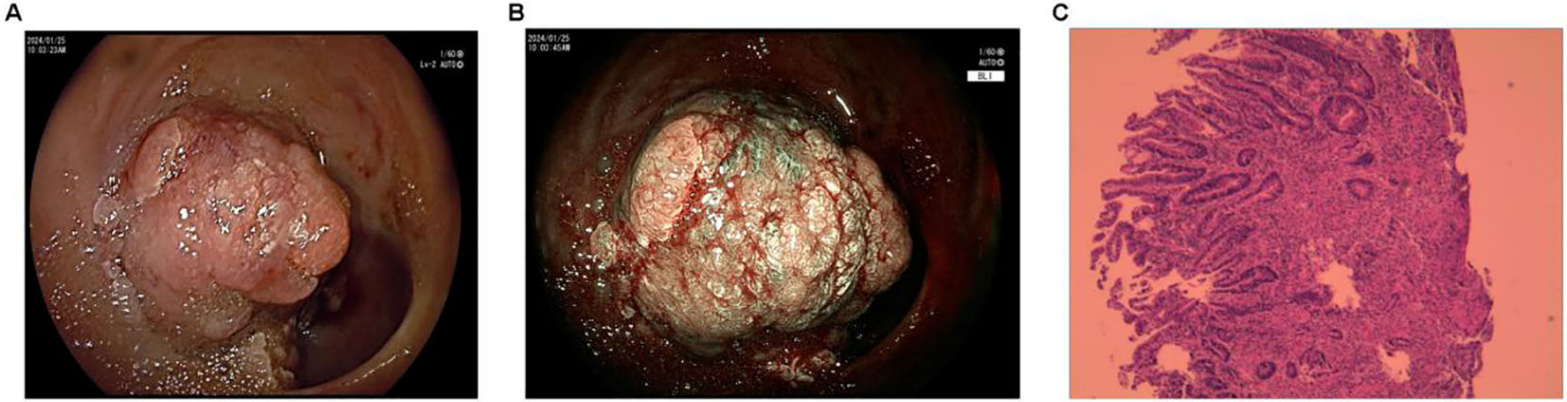
Gastroscopy and biopsy pathology. **(A)** Gastroscopy entering the duodenal stump with a bleeding tumor; **(B)** Tumor after washing away the surface blood; **(C)** Biopsy during gastroscopy showing adenocarcinoma.

Abdominal enhanced CT performed shortly after admission revealed a 31-mm-long, irregular soft-tissue mass at the proximal closed end of the duodenum in a patient with a history of subtotal gastrectomy. The mass demonstrated marked heterogeneous enhancement on post-contrast images, consistent with a hypervascular neoplasm. No evidence of regional lymph node enlargement was observed in the retroperitoneum or pelvic cavity, and no invasion of adjacent vascular structures (including the superior mesenteric artery, portal vein, and splenic vein) was identified (**Figures 2A–C**). Gastroscopy performed the following day suggested “inflammation at the anastomosis after Billroth II surgery, mass at the afferent loop” (**Figures 3A, B**); a biopsy of the stump tumor was collected during gastroscopy, and the biopsy pathology result obtained four days later suggested “Adenocarcinoma” (**Figure 3C**). Subsequent abdominal magnetic resonance imaging verified a malignant duodenal tumor with no invasion of the pancreas or biliary tract, consistent with the CT findings (**Figures 2D, E**).

### Final diagnosis

The patient was diagnosed with the following: 1. Malignant tumor of the duodenal stump(cT2N0M0, stage IIA); 2. Upper gastrointestinal bleeding; 3. Moderate anemia; 4. Postgastrectomy; 5. Cerebral ischemic foci; 6. Pulmonary shadow (pulmonary nodule); 7. Carotid plaque; 8. Hypertension; 9. Cholecystitis; and 10. Hypoproteinemia.

### Treatment

**Figure 4:**
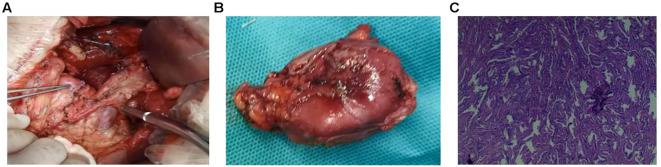
Images from surgery and postoperative pathology. **(A)** The surgical area after tumor resection and lymph node dissection, with the forceps pointing to the new stump that formed after the duodenal stump tumor resection; **(B)** The resected tumor; **(C)** Pathological diagnosis of adenocarcinoma.

Preoperatively, we provided the patient with nutritional support and completed preoperative examinations. 3 days before surgery, the patient’s hemoglobin concentration was 70 g/L, and 400 ml of packed red blood cells were transfused 2 days before to surgery. Given the patient’s advanced age and multiple underlying conditions, the risk of surgical treatment was significant. After consulting with the patient’s family, we decided on a surgical plan for duodenal stump tumor resection, with the second option being pancreatoduodenectomy. The operation was performed 13 days after admission. During surgery, we first freed the tumor and duodenum, made an incision in the duodenal wall 2 cm below the tumor, and explored to find the tumor’s lower edge, which was approximately 6 cm from the duodenal papilla; therefore, we performed duodenal stump tumor resection plus abdominal lymph node dissection (**Figure 4A**). The surgically removed specimen is shown in **Figure 4B**. Intraoperative frozen section examination revealed moderately differentiated adenocarcinoma (**Figure 4C**), with negative resection margins and no evidence of metastatic involvement in the resected lymph nodes. The final postoperative pathological diagnosis confirmed a 30 × 20 mm primary duodenal stump adenocarcinoma, moderately differentiated, with 12 regional lymph nodes negative for metastasis. The pathological stage was assigned as pT2N0M0 according to the AJCC 8th edition staging system.

## Results

**Figure 5:**
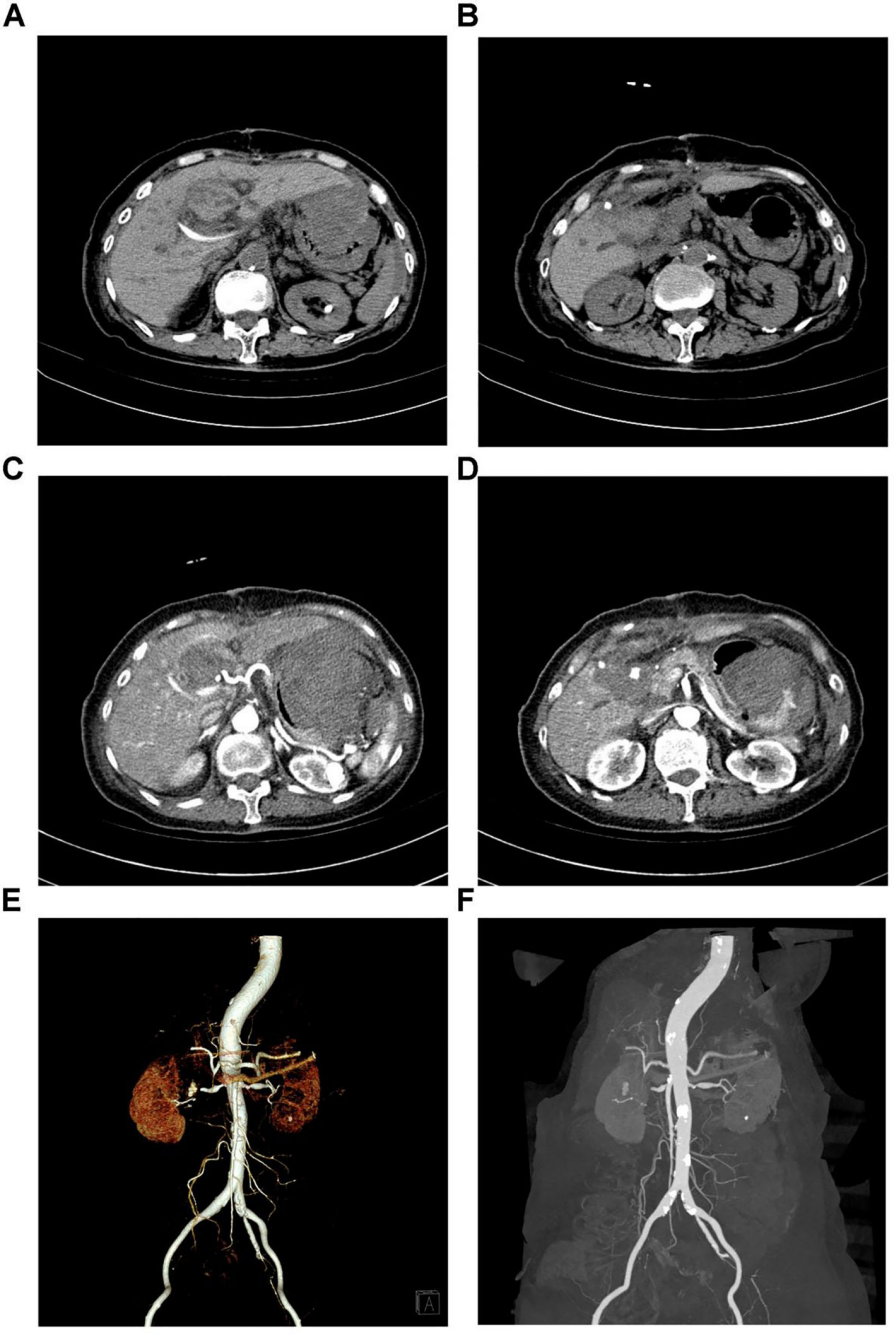
Imaging examinations during the patient’s intra-abdominal hemorrhage. **(A)** Plain CT scan revealing a hematoma between the lesser curvature of the stomach and the left liver. **(B)** Plain CT scan showing a hematoma at the porta hepatis. **(C)** Abdominal arterial CTA showing that the hematoma on the lesser curvature side and at the porta hepatis was significantly larger than revealed by plain CT. **(D)** Abdominal arterial CTA image indicating the formation of a pseudoaneurysm within the hematoma at the porta hepatis. **(E, F)** Abdominal arterial CTA vascular reconstruction revealed a pseudoaneurysm in the upper right abdomen with contrast agent leakage outside the vessels.

The patient recovered smoothly postoperatively but developed delayed intra-abdominal hemorrhage on postoperative day 11. The hemorrhage was first detected when the patient complained of right upper quadrant abdominal pain, with 80 mL of bright red blood drained from the abdominal drainage tube. Hemodynamically, the patient’s heart rate increased from 80 beats per minute to 115 beats per minute, and systolic blood pressure decreased from 135 mmHg to 90 mmHg. Abdominal examination revealed mild tenderness, predominantly in the right upper quadrant. Laboratory tests confirmed a rapid decline in hemoglobin, which dropped from 87 g/L to 69 g/L within three hours, accompanied by persistent tachycardia. Abdominal non-contrast CT was performed within 0.5 hours after the detection of hemoglobin decline and hemodynamic instability, which revealed an irregular high-density hematoma measuring approximately 5.2cm×3.1cm×3.0cm located between the lesser curvature of the stomach and the left liver (**Figures 5A, B**). Hemostatic agents including tranexamic acid injection and prothrombin complex concentrate were administered. An abdominal belt was applied for compression, and 600 mL of suspended red blood cells was transfused. Despite these measures, the patient’s hypotension and tachycardia persisted, prompting urgent abdominal vascular CTA performed 5 hours after the initial non−contrast CT.

**Figure 6:**
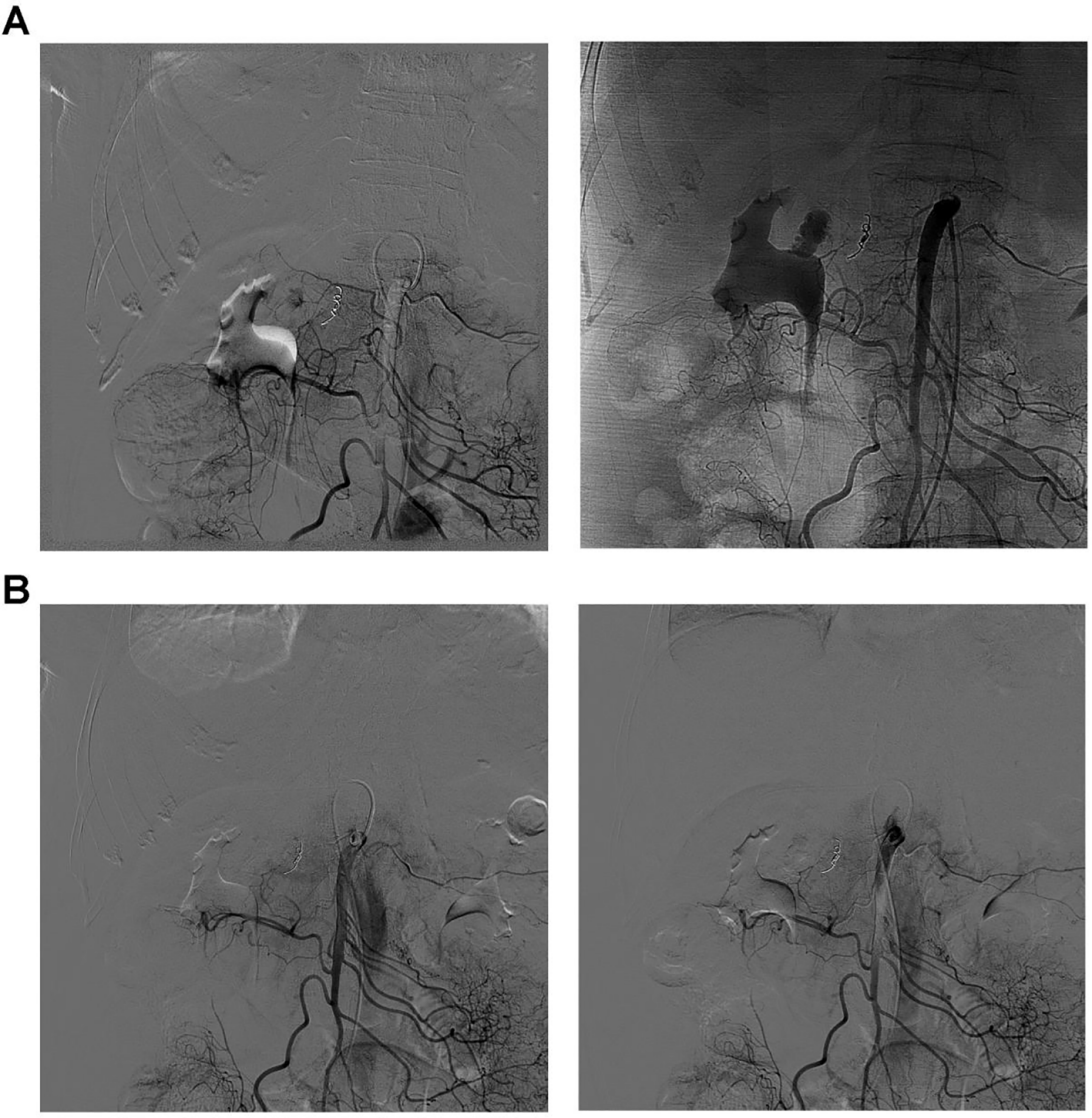
Vascular imaging before and after embolization guided by DSA. **(A)** Vascular imaging revealed bleeding from a pseudoaneurysm in a minor branch of the superior mesenteric artery. **(B)** After embolization, the area of the aneurysm showed no filling or leakage of the contrast agent.

CTA demonstrated a pseudoaneurysm arising from a branch of the superior mesenteric artery, with obvious contrast extravasation indicating active bleeding. The hematoma had enlarged to 7cm×5cm×3. cm and was located in the right upper abdomen, with bleeding extending into the abdominal and pelvic cavities (**Figures 5C–F**). Considering a rupture of the pseudoaneurysm, an emergency DSA-guided embolization of the abdominal artery was performed to stop the bleeding. Within 2 hours after the CTA diagnosis, the patient underwent emergency DSA in the interventional radiology suite. DSA clearly revealed a pseudoaneurysm measuring approximately 1.2cm×1.0cm arising from a branch of the superior mesenteric artery, which was confirmed as the bleeding source (**Figures 6A, B**). The procedure lasted approximately 1 hour, including diagnostic DSA and embolization. Superselective embolization was performed using a combination of microcoils (3 mm×2.5 mm, 4 pieces) and gelatin sponge particles. Post-embolization angiography of the superior mesenteric artery showed no filling of the pseudoaneurysm and no contrast extravasation, indicating complete hemostasis. After the bleeding was controlled, abdominal puncture and drainage were performed to reduce the symptoms of abdominal infection by draining the accumulated blood.

**Figure 7:**
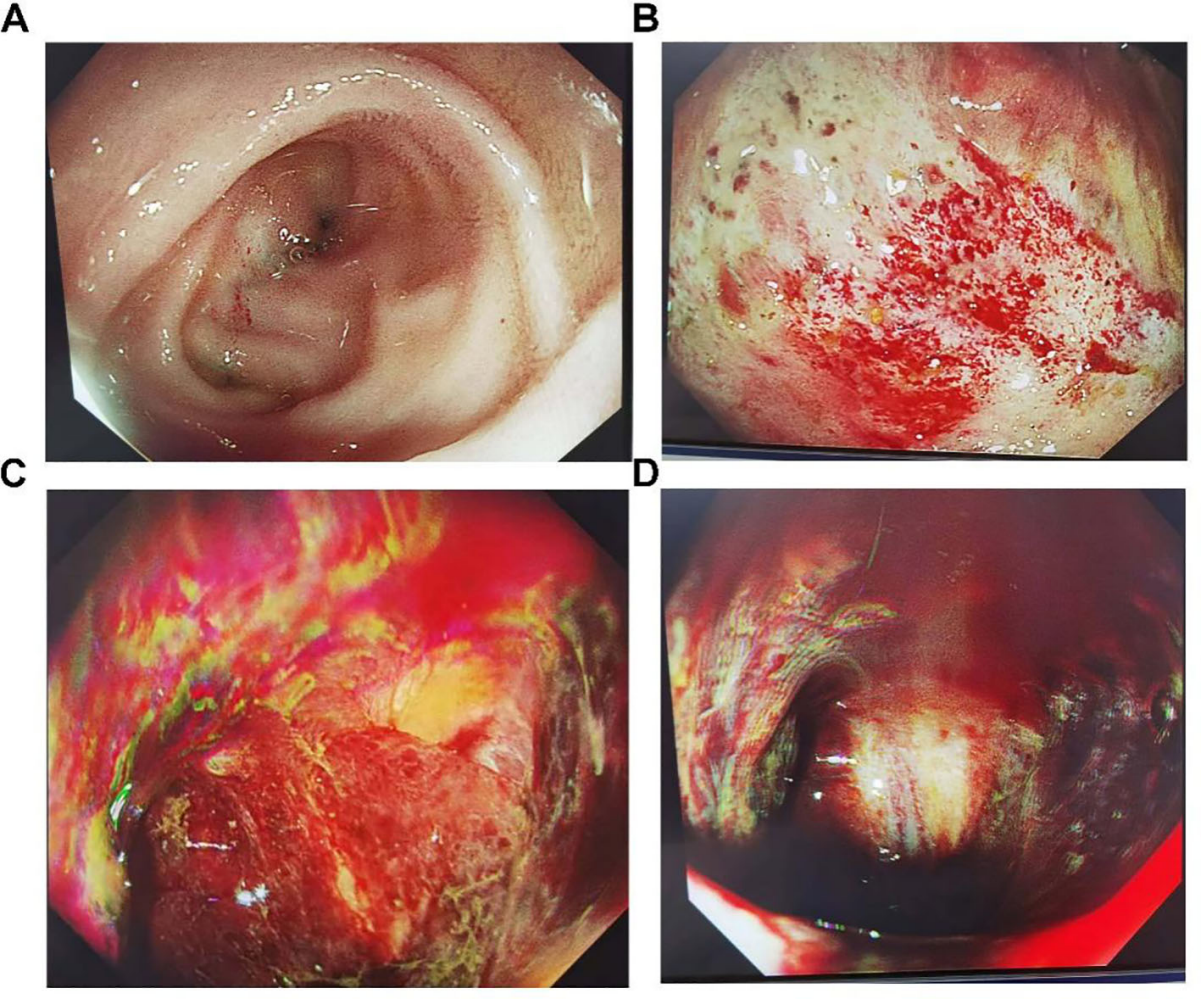
Following arterial embolization for abdominal hemorrhage guided by DSA, extensive necrosis with bleeding was observed in the descending colon, while no bleeding was observed in the duodenal stump. **(A)**. Gastroscopy revealed no bleeding at the duodenal stump. **(B–D)**. Extensive necrosis with bleeding in the descending colon.

On the 25th day after surgery, the patient developed significant symptoms of rectal bleeding, although no fluid drained from the abdominal drain. A repeat abdominal CT showed no signs of intra-abdominal hematoma. Gastroscopic and colonoscopic examinations were conducted; gastroscopy revealed no bleeding (**Figure 7A**), while colonoscopy revealed extensive ulceration with bleeding in the descending colon (**Figures 7B–D**), suggesting ischemic necrosis and bleeding due to the intestinal disease. Despite efforts to treat the condition, the rectal bleeding did not improve, the patient’s family chose to discontinue further treatment, and the patient was discharged.

## Discussion

Duodenal cancer is less common than other gastrointestinal cancers, and tumors at the duodenal stump after gastrectomy are particularly rare (1). These tumors present distinct challenges in terms of diagnosis and management (2). This case report describes a complex clinical scenario characterized by a duodenal stump tumor and significant postoperative complications (3). Duodenal stump tumors are rare and pose significant diagnostic and management challenges (4).

Duodenal stump tumors are uncommon complications following procedures such as Billroth II distal gastrectomy (5). Tumors at the duodenal stump may be primary or recurrent gastric cancer (6). Distinguishing between these tumor types is crucial because treatment approaches and prognoses vary significantly. The literature shows that duodenal cancers most often originate from the periampullary region, where the pancreatic and bile ducts enter the duodenum (7). Cancers at the duodenal stump, however, are rarer and pose distinct clinical challenges, particularly after gastric surgery (8).

In this patient with a 30-year history of Billroth II subtotal gastrectomy, distinguishing between a primary adenocarcinoma of the duodenal stump and recurrent gastric cancer was clinically pivotal, given their divergent management implications and prognostic trajectories. Several features supported the diagnosis of a primary duodenal stump carcinoma. First, the patient reported no prior history of gastric malignancy, and the original indication for gastrectomy-performed more than three decades earlier—was undocumented, rendering recurrence of a known gastric cancer unlikely. Second, cross-sectional imaging (contrast-enhanced CT and MRI) together with endoscopic evaluation consistently localized the lesion to the blind-ending duodenal limb distal to the gastrojejunostomy anatomic territory corresponding to the retained duodenal stump rather than at the anastomotic site, where recurrent gastric stump carcinoma typically arises. Third, histopathologic analysis revealed a moderately differentiated adenocarcinoma without evidence of direct extension into adjacent organs such as the pancreas or bile duct, a pattern more consistent with a tumor originating from duodenal epithelium than with aggressive local recurrence. The combination of a prolonged disease-free interval, the precise anatomic location of the tumor, and its histological characteristics collectively favors a diagnosis of primary duodenal stump adenocarcinoma. This case highlights the necessity of integrating detailed clinical history, accurate imaging and endoscopic localization, and thorough pathological assessment in the diagnostic workup of duodenal lesions in patients with remote Billroth II reconstruction.

Generally, primary duodenal tumors may be benign or malignant, with the latter being more common (9). The most frequent types are adenocarcinomas, followed by sarcomas, lymphomas, and carcinoids (10). These tumors typically result in symptoms such as obstruction, bleeding, or jaundice, influenced by their location and size. In contrast, duodenal stump tumors may remain asymptomatic until advanced stages or mimic postsurgical complications such as strictures or ulcers at the anastomosis site.

### Challenges in diagnosis and surgical management

The primary challenge in managing duodenal stump tumors stems from their anatomical location (3). The duodenum is nestled among major vascular structures and organs that complicate surgical access and increase the risk of complications (11). In patients with previous gastric surgery, adhesions and altered anatomy further complicate surgical interventions. Our patient, who previously underwent partial gastrectomy, presented with a duodenal stump tumor that was difficult to access and required intricate surgical planning (12).

The diagnosis of duodenal stump tumors is often delayed due to the subtleness of symptoms and their nonspecific nature, as was evident in this case where the patient presented with symptoms indicative of chronic anemia and intermittent gastrointestinal bleeding. The use of advanced imaging techniques such as CT and MRI plays a critical role in preoperative evaluation, allowing for a detailed assessment of the tumor’s characteristics and its relationship with surrounding structures (13).

### Surgical techniques and decision making

For our patient, the decision to perform duodenal stump tumor resection and lymphadenectomy was based on the resectability of the tumor and the absence of distant metastasis. The surgical approach was tailored to minimize disruption of the surrounding tissues and to ensure complete tumor resection. However, even with meticulous surgical techniques, the patient developed significant complications.

The occurrence of a pseudoaneurysm leading to delayed hemorrhage postsurgery is a noteworthy complication. Pseudoaneurysms after abdominal surgery are rare and can be life-threatening if not promptly diagnosed and managed. They may result from inadvertent arterial injury during surgery or from postoperative infection leading to arterial wall weakening.

### Interventional radiology in managing complications

The role of interventional radiology has become increasingly vital in managing postoperative complications such as hemorrhage. In this case, digital subtraction angiography (DSA) was instrumental in identifying the source of bleeding and facilitating targeted embolization (14). This minimally invasive technique effectively controls hemorrhage, highlighting its utility in managing complex postsurgical complications (15). However, the embolization procedure is associated with risks, including potential ischemia to the bowel, as possibly evidenced by the patient’s subsequent development of ischemic colitis (16).In this case, DSA embolization served as the probable trigger for descending colon ischemia; meanwhile, the patient had multiple critical risk factors that exacerbated ischemic severity, including advanced age, intraoperative and postoperative hypotension, and long-standing atherosclerosis involving the mesenteric vasculature. Pre-existing mesenteric atherosclerosis reduced intestinal collateral perfusion reserve, and periods of hypoperfusion further compromised intestinal blood supply, together worsening the extent of ischemic necrosis.

Furthermore, the diagnostic pathway for acute postoperative hemorrhage warrants clarification. Initial non−contrast CT provides rapid identification of intra−abdominal hematoma and guides early resuscitation, representing a valuable first step in unstable patients. Although CTA accurately localizes the bleeding source and pseudoaneurysm, excessive delay to obtain CTA may prolong time to definitive hemostasis in hemodynamically unstable individuals. In such scenarios, primary DSA without prior CTA is feasible and safe, allowing earlier embolization and improved hemodynamic control. This case underscores that a stratified strategy—non−contrast CT for initial screening, CTA for stable patients, and direct DSA for unstable patients—optimizes management of life−threatening postoperative hemorrhage.

Furthermore, comprehensive vascular assessment both on preoperative imaging and during DSA is of great importance in this clinical scenario. Preoperative evaluation of the mesenteric vasculature, including identification of atherosclerosis, anatomical variants, and collateral circulation, allows early risk stratification for postoperative bleeding and intestinal ischemia. During DSA, detailed angiographic evaluation of the superior mesenteric artery distribution and collateral pathways helps guide superselective embolization, thereby minimizing devascularization of normal intestinal segments and reducing the risk of post-embolization ischemic complications. Routine systematic vascular assessment may facilitate prediction of pseudoaneurysm formation and enable preventive strategies, ultimately improving the safety of endovascular hemostasis in high-risk surgical patients.

### Vascular risk factors for postoperative complications

The patient was a 77-year-old female with a 20-year history of hypertension and pre-existing atherosclerosis of the abdominal aorta and superior mesenteric artery as demonstrated on preoperative imaging. Advanced age, long-standing hypertension, and underlying mesenteric atherosclerosis may weaken arterial wall integrity, predisposing to localized vascular injury during dissection and subsequent pseudoaneurysm formation. Furthermore, pre-existing sclerotic changes likely reduced the collateral perfusion reserve of the intestinal circulation, increasing the risk of ischemic colitis after superselective embolization of the superior mesenteric artery branch. These vascular comorbidities represent important, underrecognized contributors to the severe postoperative course in this case.

## Conclusion

Duodenal stump tumors present significant surgical challenges, particularly in patients with a history of major gastric surgeries. While surgical resection remains the primary treatment modality, the potential for severe complications, such as delayed intra-abdominal hemorrhage, necessitates careful consideration. This case exemplifies the critical need for a thorough preoperative assessment, meticulous surgical technique, and postoperative vigilance to promptly identify and manage complications. Interventional radiology is indispensable in the acute management of postsurgical complications but requires careful handling to avoid further complications such as ischemia. Further studies are needed to develop guidelines to optimize surgical approaches and postoperative care for patients with similar presentations to improve outcomes and reduce the risk of life-threatening complications.

## Data Availability

The original contributions presented in the study are included in the article/supplementary material. Further inquiries can be directed to the corresponding author.
